# Predicting Collective Action in a Secessionist Context: Different Motives for Two Opposed Stances

**DOI:** 10.3389/fpsyg.2021.700530

**Published:** 2021-08-04

**Authors:** Marcos Dono, Monica Alzate, José-Manuel Sabucedo

**Affiliations:** ^1^Cross-Disciplinary Research in Environmental Technologies (CRETUS), University of Santiago de Compostela, Santiago de Compostela, Spain; ^2^Department of Social, Basic and Methodological Psychology, Faculty of Psychology, University of Santiago de Compostela, Santiago de Compostela, Spain

**Keywords:** Catalonia, collective action, moral obligation, nationalism, secession

## Abstract

Engagement in collective action is essential in the scenario of a secessionist struggle. In this scenario, two groups contend for an incompatible goal and one of them is favoured by the current status quo. Therefore, this context represents an excellent opportunity to compare the motives for participation among two groups whose situation and objectives differ drastically. We examined the motivations to participate in collective action of Catalan participants in the days leading to the independence referendum held in Catalonia (Spain) on the first of October 2017 (*n* = 719). As hypothesized, participation predicted by different motivations for each group. Regarding participation in the referendum, Catalan identity was the only predictor among pro-independence ranks, while those against independence showed a solidarity-based motivation. This work contributes to the literature by adapting previously researched collective action motivations to the context of a secessionist contention and providing evidence of their effect. Crucially, the motivations are different between supporters and opponents of independence, highlighting the need for examining the status and the stance on the system of groups when studying collective action.

## Introduction

Despite a longstanding strong identification with an own Catalan culture and costumes, the movement for Catalan independence only started to gain strength in 2012 (Martí, 2013; Qvortrup, 2021) and reached its zenith years after, in the month of October 2017. Then, after an independence referendum that was previously declared illegal by the Spanish Constitutional Court with the support of the Venice Commission, Catalonia briefly declared its independence on October 27. The declaration was immediately suspended and never took full effect. However, these events had a tremendous impact that still to this day divides the Catalan Society.

Secessionist struggles are among the political confrontations with a greater potential impact for the population. Secessions can impact the economy of both the region that contends for independence and the host state (Thomas, 2018). It can also affect future international relations (Qvortrup, 2014; Reuters, 2017). Even more impactfully, disputes for independence can generate intergroup conflict as society becomes rent asunder by two incompatible governing models. This could ultimately lead to violence, as shown by the relatively recent example of the Irish Troubles (Bell, 1993). The conditions as well as the political strategies and narratives that characterize the emergence and success of secessionist movements have been extensively analysed (Hechter, 1992; Qvortrup, 2014; Cetrà and Liñeira, 2018; Arrighi, 2019; Sabucedo et al., 2020). Yet, despite the crucial role collective action has in this scenario, research on psychosocial collective action motives in the context of a secessionist struggle is scarce (for a notable exception, see Abrams et al., 2020).

This study examined the motivations for participation in collective action in favour and against independence and the 1-O referendum in the days before the voting. It is important to clarify that the referendum will be considered as a protest in order to analyse the motivations leading to participation. We consider this to be the best approach as the circumstances surrounding the referendum disqualify it as a regular voting for several reasons: the referendum was declared illegal by the Constitutional Court, the Venice Commission did not endorse the referendum and the control of the voting process (electoral registers, voter identification and counting of votes) was performed by the organizers rather than an impartial body (BBC, 2017a; Gómez, 2017). Moreover, the will of the Spanish Central Government was to not only ignore the result but also avoid the voting taking place at all, as police was deployed to try to stop the event (Mateo, 2017; Qvortrup, 2021). This scenario would cast, at a minimum, serious doubts among voters that the result of the plebiscite would materialize, and would undoubtedly discouraged the participation of opponents of independence (Arrighi, 2019).

Aside from examining the motivations leading to participation in collective action in a secessionist context, this research represents an excellent opportunity to compare such motivations for two groups with opposed goals: one of them system challenging and the other system supportive. In this sense, the pro-independence group represents a system-challenging position as they wish to change the current configuration of the Spanish state, while the unionist group could be labelled as system supportive. Not only the pro-independence group wishes to achieve a systemic change, but also several authors have argued that they were able to re-channel the previous system-challenging discourse that arouse after the 2008 economic crisis to build a case against the Spanish state and in favour of Catalan independence (Sabucedo et al., 2017; Miley, 2019). However, it is important to notice that the pro-independence group was in various respects privileged inside Catalonia. First and foremost, the Catalan Government had been pro-independence for several years at the time of research (Qvortrup, 2021) but research also shows that the pro-independence camp in Catalonia is in overall terms of a higher socioeconomic status (Miley and Garvía, 2019; Oller et al., 2020). Ultimately, this asymmetry in the situation and aims of both groups should imply that different motivations influence action in a distinct fashion (Jost et al., 2017). Previous to the 1-O referendum, only the system-challenging group had numerously and repeatedly demonstrated (Burgen, 2012; Kassam, 2015; Reuters, 2016; Minder, 2017). On the other hand, those against independence rarely engaged in collective action. A circumstance that changed only after the 1-O referendum, when there was a surge of anti-independence mobilizations (BBC, 2017b).

### Motives for Collective Action Participation in Secessionist Contexts

To this day, three constructs have garnered the most empirical support as motivations for collective action: identity, injustice and efficacy (Gamson, 1992; Sturmer and Simon, 2004; van Zomeren et al., 2008; Tausch et al., 2011). More recently, other motives have been integrated into explanatory models of collective action. Especially, notable has been the incorporation of moral obligation (Vilas and Sabucedo, 2012; Alberici and Milesi, 2018; Sabucedo et al., 2018, 2019). Yet these variables may not work straightforwardly in the context of secession, as the nature of the conflict confronts a group that aims to support the current system with one that seeks to challenge it. This is a crucial difference that should influence the importance of each motivation in driving participation (Jost et al., 2017). It also implies that some of the motives may have to be reformulated for them to be fully effective. Hereunder, the anticipated impact of the motives for each stance on independence is discussed.

In the context of a secessionist movement, the main social identities confronted are regional and national (Hechter, 1992; Abrams et al., 2020). Furthermore, identification with both groups – Catalonia and Spain – is possible in this context, most likely among those against independence. Hence, attitudes and behaviour might be determined by people’s degree of identification with both groups. In this sense, someone that strongly identifies with both groups will develop what is labelled as a dual identity and would be deterred to act in ways that are judged to be detrimental for one of such identities (González and Brown, 2006). A testament to the impact of this dual identification is laid up by Arrighi (2019) as he notes that out of all the independence referendums held worldwide between 1980 and 2016, the only three where the option against independence prevailed took place in ‘plurinational democracies’. This is exactly the case of Catalonia, which since the re-establishment of Spanish democracy has enjoyed a high degree of self-government (Liñeira and Cetrà, 2015; Qvortrup, 2021).

Thus, exclusive identification with Catalonia should promote intention to participate for the pro-independence group, while Spanish and dual identities should have the same effect for people who stand for the unity of Spain. Lack of identification with Spain is anticipated to heighten intention to participate among those in favour of independence as it impedes dual-identification processes (González and Brown, 2006), while identification with Catalonia is anticipated to not be a significant predictor among anti-independence ranks. The reason is that, in a case of dual identity, people against independence would not perceive defending the permanence of Catalonia within Spain as an act that may hinder their Catalan identity as both are integrated in the superordinate Spanish identity.

Regarding injustice, research on collective action participation dynamics showed that, over and above considerations on objective conditions, it was the subjective sense of collective deprivation that consistently predicted engagement in collective action (Gurr, 1970; Gamson, 1992). Later research has shown that the emotional dimension of this relative deprivation, labelled affective injustice, emerges a superior predictor of collective action when compared to its strictly cognitive counterpart (van Zomeren et al., 2008). Negative emotions will then be assessed to represent injustice. However, typical group-based affective injustice measurements may not be comparable in this context, where one group is clearly favoured by the current situation. Since the injustice frame has an adversarial character (Gamson, 1992), injustice has been conceptualized as negative emotions towards etiher the Catalan or Spanish Goverment. This conception of injustice appears as especially useful in the Catalan context, where a political climate of severe confrontation between the pro-independence and the unionist camps has emerged in recent years (Arrighi, 2019; Oller et al., 2019a). Collective action is anticipated to be predicted by negative emotions against the Spanish Government for those in favour of independence, and against the Catalan Government for those against secession.

Efficacy is another motivation that may not be directly comparable. People against independence are favoured by the current state of things; therefore, their goal would be to maintain the situation as it is. On the other hand, pro-independence groups seek radical change. Hence, we believe that it may be misleading to compare their levels of perceived efficacy towards a specific goal, despite this being the most common assessment of group efficacy in the literature (van Zomeren et al., 2008). Alternatively, efficacy is measured using positive emotions, such as hope and optimism as a proxy. These emotions that have been observed to be related to efficacy perceptions and to lead participation (Just et al., 2007; Sabucedo and Vilas, 2014; Wlodarczyk et al., 2017; Cohen-Chen and Van Zomeren, 2018).

Crucially, a positive emotional climate has recently been found to promote participation in the context of Catalan pro-independence movements through appealing to efficacy perceptions and the belief in a better future (Sabucedo et al., 2017). However, hopeful perspectives of the future are more likely to predict participation specifically for those who seek change. Conversely, those against independence seek to maintain a status quo that, under current circumstances, they are likely to perceive as being threatened. For these people to perceive a positive emotional climate would at least mean seeing pro-independence claims as legitimate. Thus, perception of a positive emotional climate should be related to increased intentions to participate for those in favour of independence but not for those against.

Finally, it is necessary to address the role of moral obligation (Vilas and Sabucedo, 2012; Alberici and Milesi, 2018; Sabucedo et al., 2018). Moral obligation comprises a series of beliefs and moral emotions that motivate people to behave accordingly with one’s moral self-guides (Sabucedo et al., 2018). Rather than reflecting the possession of a given belief or value, moral obligation represents the intensity with which people feel compelled to act upon those values. Crucially, moral obligation represents a personal motivation for behaving accordingly with one’s moral values independently of what other agents may think (Sabucedo et al., 2018). For this reason, it is thought it will equally predict intention to participate across both groups.

Despite having been framed as a protest, participating in the Referendum should be predicted by different motives for each group. In the case of the pro-independence group, it is expected that it will be predicted by the same motives that lead to a general intention to participate in collective action. However, this is not expected to be the case for those against independence. This is since participating in the referendum – especially when it has been declared illegal – is incongruent with defending the current state configuration. Therefore, voting in the referendum should be interpreted as support for the groups that defend being able to vote on this issue, and not an endorsement of that cause. Previously, collective action in solidarity with another group has found to be motivated by anger towards the group favoured by the status quo (Saab et al., 2015). In this case, we anticipate that feeling aggrieved by the management of the Spanish Government on the Catalan situation will predict intentions to participate in the Referendum as an act of protest claiming for a different approach on the situation. Additionally, we believe that for people against independence to participate in the –previously declared illegal – 1-O Referendum, they should judge the actions of the Catalan Government as legitimate. Hence, the intention to participate in the Referendum by this group should be enhanced by the lack of negative emotions towards the Catalan Government.

Based on the previous arguments, we present the following hypotheses:

*H1*: The *pro-independence* group has a higher intention to participate in both collective action in favour of the independence and the referendum.

*H2*: For the *pro-independence* group low identification with Spain, high identification with Catalonia moral obligation, positive emotional climate and negative emotions towards the Spanish Government result in heightened intention to participate in collective action, including the 1-O referendum.

*H3*: For the *anti-independence* group identification with Spain, moral obligation and affective injustice are related to a stronger intention to participate in collective action in favour of the unity of Spain.

*H4*: For the *anti-independence* group, negative emotions towards the Spanish Government and low negative emotions towards the Catalan Government drive participation in the 1-O referendum.

## Materials and Methods

### Procedure and Participants

The present research was approved by the University of Santiago de Compostela Bioethics Committee. Following the indication of Schönbrodt and Perugini (2013), a minimum of 250 participants are needed to achieve stable correlations. Thus, the aim was to recruit at least 300 participants from each group (in favour or against independence) to prevent falling short due to missing data. The study was administered as an online survey, and the final sample was composed of 719 Catalan participants (65% women; *M*_age_ = 33.61; *SD* = 8.58). 300 participants declared to be against independence, while 419 claimed they were in favour. The questionnaires were adapted to participants’ position about independence. For example, when asked about moral obligation and their intention to participate in political protests, participants in favour of independence were asked about fighting for independence. Alternatively, those against independence were enquired about defending the permanence of Catalonia as a Spanish region.

### Measures

*Stance on independence* was measured by a single item asking whether people were in favour or against the independence of Catalonia.

#### National Identity

Participants indicated how much they identify with both Spain and Catalonia by signalling how much their ‘self’ overlapped with the Spanish and Catalan flags in a single-item pictorial measure based on the work of Aron et al. (1992).

#### Negative Emotions Towards the Adversary

Participants stated how they felt when they thought about the Spanish and Catalan Governments. Two emotions were assessed: *anger* and *outrage*. Items ranged from 1 (nothing) to 7 (very much). Reliability was assessed using Cronbach’s alpha (*α* = 0.81 against Spanish Government; *α* = 0.91 against Catalan Government).

#### Positive Emotional Climate

Participants indicated how they perceived the emotional climate of the Catalan society to be positive at that moment by responding to three 7-point Likert items (*hope, joy and solidarity*) form the emotional climate scale by Páez et al. (1997; *α* = 0.83).

#### Moral Obligation

Participants indicated their agreement with statements indicating how morally obligatory was it to engage in certain actions to defend their position about independence (*e.g. to mobilize in favour of Catalonia’s independence/permanence of Catalonia in Spain constitutes a moral obligation to oneself*). Moral obligation was measured with 5 items ranged from 1 (totally disagree) to 7 (totally agree; *α* = 0.90; Sabucedo et al., 2018).

#### Intention to Participate in Collective Action

Participants indicated how prone they were to participate in different acts of political protest to defend their position regarding Catalonian independence (e.g. demonstrating, going on strike). The three items ranged from 1 (completely disagree) to 7 (completely agree; *α* = 0.89; Sabucedo and Arce, 1991).

Finally, a single-item measure was used to assess *participation in the 1-O Referendum* (‘Will you participate in the 1-O Referendum?’).

## Results

A first preliminary analysis of the data was performed by comparing the means of both groups in all introduced variables. A factor ANOVA was performed using stance on independence as the factor variable (see Table 1). Scores for moral obligation, positive emotional climate and negative emotions towards the rival government were all lower for the anti-independence group, as well as intention to participate in both collective action and the referendum. These results are congruent with the greater commitment and emotional activation that an action against the status quo, such as independence, requires.

**Table 1:** Results of intergroup comparisons for participation motives variables using factor ANOVA (*n* = 719).

	*M*(*SD*)		*F*(1)
	Pro-independence (*n* = 419)	Anti-independence (*n* = 300)	
Spanish identity	1.73 (0.80)	3.36 (1.34)	409.7^*^
Catalan identity	4.09 (1.04)	3.23 (1.27)	96.2^*^
Negative emotions towards Spanish Government	5.28 (1.55)	3.83 (1.90)	126.5^*^
Negative emotions towards Catalan Government	1.69 (0.88)	3.99 (1.98)	436.5^*^
Positive emotional climate	4.01 (0.62)	2.57 (0.97)	579.2^*^
Moral obligation	4.95 (1.33)	3.62 (1.87)	124.5^*^
Intention to participate in collective action	5.90 (1.30)	3.28 (1.80)	506.1^*^
Intention to participate in the 1-O referendum	0.88 (0.32)	0.31 (0.46)	383.0^*^

* *p* < 0.001.

Referendum participation is dummy-coded 0–1 (no participation-participation).

### Intention to Participate in Collective Action

To explore the motives leading to participation for both actions, data were split based on stance on independence or unity of Spain, and afterwards, all collective action motives were regressed on the *intention to participate in collective action*.

The results for the pro-independence group are presented in Table 2, and the following variables turned out to be significant: low identification with Spain, high identification with Catalonia, positive emotional climate, negative emotions towards the Spanish Government and moral obligation. The proposed model yielded an effect size of *f*^2^ = 0.39, which according to (Cohen, 1988) benchmark represents a large effect size.

**Table 2:** Results of linear regression analysis for intention to participate in collective action in the pro-independence group (*n* = 419).

	*B* [95% *CI*]	*SE*	*β*	*p*	VIF
Spanish identity	−0.20 (−0.34, −0.06)	0.07	−0.12	0.005	1.19
Catalan identity	0.13 (0.02, 0.25)	0.06	0.11	0.026	1.36
Negative emotions towards the Spanish Government	0.11 (0.04, 0.18)	0.03	0.13	0.003	1.11
Negative emotions towards the Catalan Government	0.04 (−0.08, 0.16)	0.06	0.02	0.537	1.15
Positive emotional climate	0.36 (0.17, 0.55)	0.09	0.17	<0.001	1.29
Moral obligation	0.27 (0.18, 0.37)	0.04	0.28	<0.001	1.49

*F*(6) = 27.9, *p* < 0.001, Adjusted *R*^2^ = 0.28.

The regression analysis for the anti-independence group is displayed in Table 3. As hypothesized, moral obligation and Spanish national identity were significant predictors of collective action intention. It is also significant to point out that in this group, motivations to participate are based neither on the rejection of Catalonian identity nor on negative emotions towards the Catalan Government. This regression analysis also showed a large effect size (*f*^2^ = 0.53).

**Table 3:** Results of linear regression analysis for intention to participate in collective action in the anti-independence group (*n* = 300).

	B [95% *CI*]	*SE*	*β*	*p*	VIF
Spanish identity	0.18 (0.01, 0.36)	0.09	0.14	0.043	2.13
Catalan identity	0.04 (−0.11, 0.20)	0.07	0.03	0.564	1.40
Negative emotions towards the Spanish Government	−0.02 (−0.13, 0.09)	0.05	−0.02	0.746	1.55
Negative emotions towards the Catalan Government	0.04 (−0.06, 0.15)	0.05	0.05	0.451	1.74
Positive emotional climate	0.10 (−0.12, 0.32)	0.11	0.05	0.378	1.71
Moral obligation	0.48 (0.36, 0.60)	0.06	0.50	<0.001	1.87

*F*(6) = 27.9, *p* < 0.001, Adjusted *R*^2^ = 0.35.

### Intention to Participate in the Independence Referendum

The same motives previously regressed onto intention to participate in collective action were regressed on the intention to participate in the independence referendum that took place the first of October 2017. Since the intention to participate in the referendum was measured binomially, the chosen analysis was binary logistic regression. The model for the pro-independence group showed a correct classification index of 88.1%. Only Catalan identity provided a significant odds-ratio (see Table 4).

**Table 4:** Results of binary logistic regression analysis for intention to participate in the 1-O referendum in the pro-independence group (*n* = 419).

	*B*	*SE*	Exp(*B*) [95% *CI*]	*p*
Spanish identity	−0.36	0.20	0.69 (0.46, 1.03)	0.073
Catalan identity	0.71	0.15	2.03 (1.49, 2.77)	<0.001
Negative emotions towards the Spanish Government	−0.21	0.11	0.80 (0.64, 1.01)	0.053
Negative emotions towards the Catalan Government	−0.09	0.16	0.91 (0.66, 1.24)	0.568
Positive emotional climate	0.14	0.28	1.15 (0.65, 2.02)	0.627
Moral obligation	0.10	0.13	1.10 (0.84, 1.44)	0.466

*χ*^2^ = 38.99, *p* < 0.001, Nagelkerke’s Pseudo-*R*^2^ = 0.17.

As for the anti-independence group, the model’s correct classification index amounted to 74.3%. In this case, the model yielded three significant odd-ratio values. Low Spanish national identification, low negative emotions towards the Catalan Government and high negative emotions towards the Spanish Government all promoted participation in the referendum (see Table 5).

**Table 5:** Results of binary logistic regression analysis for intention to participate in the 1-O referendum in the anti-independence group (*n* = 300).

	*B*	*SE*	Exp(*B*) [95% *CI*]	*p*
Spanish identity	−0.37	0.16	0.68 (0.50, 0.93)	0.021
Catalan identity	0.14	0.16	1.15 (0.84, 1.58)	0.365
Negative emotions towards the Spanish Government	0.35	0.10	1.42 (1.17, 1.73)	<0.001
Negative emotions towards the Catalan Government	−0.39	0.10	0.67 (0.55, 0.83)	<0.001
Positive emotional climate	0.30	0.19	1.34 (0.93, 1.95)	0.115
Moral obligation	0.04	0.10	1.04 (0.84, 1.28)	0.684

*χ*^2^ = 84.59, *p* < 0.001, Nagelkerke’s Pseudo-*R*^2^ = 0.34.

## Discussion

The present paper sought to test the psychosocial motivations leading to collective action participation in a secessionist context. To achieve this, data were gathered in Catalonia in the days prior to the 1-O independence referendum in 2017. The first contribution of the present work is that the motivations hereby examined remain relevant in a context of secessionist struggle, replicating findings from previous research that used these motives in other contexts (van Zomeren et al., 2008; Sabucedo et al., 2018). This represents a novel contribution as some of the collective action frames have been adapted for the specific case of a secessionist struggle.

Yet, the main objective of this research was to test whether these two groups differed in their motivation to participate. This was indeed the case, as the motivational setting between groups in favour and against independence differed: while all motivations were significant predictors for those in favour of independence, some of them failed to drive those against secession to participate. As hypothesized, the perception of a positive emotional climate revolving around hope in the future did predict collective action for the pro-independence group, but not the anti-independence group. These positive emotions may be the result of having the Catalan regional government, and all its resources in favour of the independence cause and the perception that the Spanish Government would end up giving in in that confrontation (Sabucedo et al., 2017). On the contrary, non-independence people were more concerned about the tension that was being experienced and the one that could come. These results dovetail previous findings about the impact of positive affect in driving political participation in a secessionist context (Sabucedo et al., 2017; Oller et al., 2019b).

More surprising is the fact that motivational schemas also differ on the significance of negative emotions towards the adversary, as the anti-independence group is not motivated by negative emotions against the Catalan Government. A potential explanation for this finding may be that the effect of group-based anger is moderated by a perception of threat to the status quo. Therefore, anger will not be sufficient to promote system-supportive collective action by a system-supportive group if that group perceives the acts of the rival group as non-threatening to the current status quo. Moreover, the overall level of anger against the Catalan Government shown by unionists (*M* = 3.99, *SE* = 1.98) was much lower (and only slightly over the midpoint of the scale) than the anger level that the pro-independence camp showed against the Spanish Government (*M* = 5.28, *SE* = 1.55). Both the argument and the findings are coherent with the role of anger as an emotional response to threat (Neuman et al., 2007; Neuberg et al., 2011; Marcus et al., 2019). Also endorsing the thesis hereby posited, recent research has shown that groups perceiving threats against them feel more anger, which leads them to a higher willingness to participate (Gutierrez et al., 2019). The capital role of threat has also been attested in qualitative research, where it has been defined by activists as a ‘pre-condition to get up from the couch’ (Kleres and Wettergren, 2017, p. 512). Also supporting this argument is the fact that the first massive demonstration against independence took place only after the 1-O referendum and the brief declaration of independence that followed (BBC, 2017b) when the threat would seem more obvious.

The final difference found in the motivational configuration of the groups refers to the identity variable. Pro-independence collective action is predicted positively by national Catalan identity and negatively national Spanish identity. On the other hand, participation among those against independence is only predicted – positively – by identification with Spain. This supports the hypothesis that action supportive of secession will be deterred by a dual-identification process, as the success of such actions will be detrimental for one of the two identities (González and Brown, 2006).

Finally, moral obligation did predict collective action for both groups. This finding highlights the importance of moral obligation as a predictor of collective action (Vilas and Sabucedo, 2012; Sabucedo et al., 2019), and it also supports the idea that moral concerns equally motivate behaviour regardless of specific beliefs (Schein and Gray, 2017; Dono et al., 2018).

Regarding participation in the referendum, the results did not support our hypotheses in what refers to the pro-independence group. Participation on the referendum was conceived as a collective action and thus was anticipated to be explained by the same variables that should predict intention to participate. However, only identification with Catalonia significantly predicted voting for pro-independence people. This is caused by a ceiling effect that may respond to the perception of the referendum as a ‘*once in a lifetime*’ opportunity. Another contributing factor may be the recent example of Scotland, where the victory of the permanence in the United Kingdom was then argued to settle the secessionist conundrum.

The results concerning participation in the referendum by anti-independence group partially supported our hypothesis. This is so as participation was predicted by negative emotions towards the Spanish Government and by low negative emotions towards the Catalan Government, but also – unexpectedly – by low identification with Spain. We believe this profile reflects an individual that is discontent with the treatment that the Spanish Government has given to the situation. Therefore, participating in the referendum is conceived as a solidary symbolic act to claim for a different management of the situation. Results also manifest that the voting was not framed as a binding referendum for the anti-independence group, as participation was not driven by a will to defend the current state configuration (i.e. moral obligation). The findings about the motivation of anti-independence people to participate in the referendum also replicate the role of negative emotions as an important motive for solidarity-based collective action (Saab et al., 2015).

### Shortcomings and Future Directions

The variables included in our survey were able to generate significant predictive models of people’s participation in collective action and the 2017 1-O referendum, providing relevant information about people’s psychosocial underpinnings to participate in such events. However, our study also displays some limitations. Importantly, the sample had young adults overrepresented. Additionally, although required by the very nature of the study, the research was performed exclusively with Spanish/Catalan participants, which raises questions on the degree of generalizability of the results.

Considering future research, other variables may be worth exploring as motives for collective action in this context. For instance, something that may shape intentions to participate in such a context is the perception of third-party support. The success of secessionist movements has been deemed to depend on third-party acknowledgement (Hechter, 1992; Heraclides, 2012; Buchanan, 2017). However, to the best of our knowledge, this has not been examined as a motivation for collective action. Are people in a secessionist struggle encouraged to participate in collective action by perceived third-party support as it heightens efficacy feelings? Or are they driven by the perception of lack of support as it makes injustice more acute? This seems an interesting avenue to explore when it comes to political participation, even outside of a secessionist context.

Finally, we would like to suggest another potential avenue for research regarding secessionist collective action. Secession ultimately means taking a very drastic action through which one’s in-group is detached from a previous superordinate group in which it was formerly included. According to this logic, we believe that a potential explanation for the arising of secessionist attitudes can be explained through the quest of significance. This construct is defined as the aspiration people have to matter and be relevant within their social environment (Kruglanski and Orehek, 2011). It is hereby proposed that secessionism could be a strategy to fight feelings of irrelevance, as splitting from the previous superordinate identity will confer the opportunity to define the new, seceded identity in specific terms that may reassure personal worth and purpose.

## Conclusion

In the present work, some of the most empirically supported motivations for participating in collective action (van Zomeren et al., 2008; Sabucedo et al., 2019) have been successfully adapted to compare the potential engagement of two groups in secession-related collective action. Particularly, the motivations of identity, efficacy and injustice have been conceptualized in a different fashion than usual, seeking to guarantee an optimum comparison between the groups. For the most part, the results support the hypotheses formulated, including those regarding the different motivations for collective action participation in each group. Secessionist struggles provide a paradigmatic case of two groups with opposite goals, where one of them wants to maintain the status quo, while the other seeks to challenge it.

Despite political debates around most issues confront system-challenging and system-supportive positions, only recently scholars have called for an examination of this circumstance (Jost et al., 2017). The approach by Jost et al. (2017) describes how system justification influences the motives that lead system-supportive and system-challenging members to collective action participation and has recently gathered empirical support (Osborne et al., 2019). The present research extends these findings as it shows that, under certain circumstances, some motivations may work only for one of such groups, as it is the case of affective injustice and positive emotions in this research. It seems that dual-identity groups that support the current juridic configuration of the state may need to perceive an evident threat to either that configuration or their social identity for their grievances to drive them into action. Besides, the fact that they are already in the desired position nullifies the motivating effect that positive emotions may have. The only motivations that remain significant predictors for the system-supportive group are identity and moral obligation. Hence, individuals highly committed with their group and those driven by moral concern seem disposed to participate in any circumstance. This supports the argument of both these motivations being specially important driving factors for participation (van Zomeren et al., 2008; Sabucedo et al., 2018).

Contentions for secession have great potential implications for society and pose the risk of stagnating and even becoming violent conflicts. Thus, a great deal of attention needs to be directed at seeking a satisfactory and peaceful solution to these conundrums. Understanding how and why collective action motivations differ between the groups in contention is a key part of reaching such a solution, but it should also be applied to other political controversies. Politics usually confront those who seek change against those who resist it: neglecting how these opposing stances shape their motivation to participate in collective action is bound to produce imprecise knowledge.

## Data Availability Statement

The raw data supporting the conclusions of this article will be made available by the authors, without undue reservation.

## Ethics Statement

The studies involving human participants were reviewed and approved by Bioethics Committee of the University of Santiago de Compostela. The patients/participants provided their written informed consent to participate in this study.

## Author Contributions

All authors listed have made a substantial, direct and intellectual contribution to the work, and approved it for publication.

## Conflict of Interest

The authors declare that the research was conducted in the absence of any commercial or financial relationships that could be construed as a potential conflict of interest.

## Publisher’s Note

All claims expressed in this article are solely those of the authors and do not necessarily represent those of their affiliated organizations, or those of the publisher, the editors and the reviewers. Any product that may be evaluated in this article, or claim that may be made by its manufacturer, is not guaranteed or endorsed by the publisher.
